# Tracheotomy and Epilepsy

**Published:** 1854-12

**Authors:** 


*Tracheotomy and Epilepsy.*—The last number of Ranking's Abstract gives five cases of this operation, two of which afterward died during the epileptic seizure.

When Dr. Marshall Hall proposed this operation, he risked a good deal upon a theory. A glance at the subject shows that it was an operation directed, not to the disease, but to one of its symptoms—laryngismus. The discussion which this operation has occasioned, has elicited some facts of importance in the symptomatology of epilepsy, relative to the frequency of laryngismus.

Cases of epilepsy occur (and it is in these that Dr. Hall's operation seems justifiable) in which the laryngeal spasm is a prominent symptom, producing temporary asphyxia with a corresponding determination of blood to the brain, which must prove extremely mischievous to the integrity of that organ. It seems that in this case the convulsion is primarily in the larynx, and that the violent action of the muscular system is dependent upon it. Here the operation of tracheotomy may come in between a symptom and its consecutive disturbances, and reduce the attack to a simple loss of consciousness for the moment, without spasm, making the case one of *petit*, instead of *grand mal.*

There are other cases in which the function of respiration is not at all, or very little, impaired; both the laryngeal and thoracic muscles continuing their normal action. In these cases no benefit could be expected from opening the trachea.

In still another variety of cases there exists a spasm, more or less tonic, of the thoracic muscles, the diaphragm, intercostals, and external thoracic muscles being in a state of rigidity, or else of clonic spasm, acting without harmony or consent, so as to equally impair the respiratory function. The patient may die from asphyxia, but not from closure of the trachea. The walls of the chest, as in tetanus, refuse by their rigidity to admit air. Here, also, tracheotomy would be useless.

These reflections narrow down the usefulness of this operation to a very limited number of cases, and as might be expected, even in these cases it has happened that in subsequent seizures the thoracic muscles have become fixed, and the patient has died of asphyxia with a tube in his windpipe. H.

---

We publish the following with pleasure:

*Drs. Flint and Hunt:* Sirs,—In the September number of your excellent "Journal," I see an editorial on Spirit Rappings, which precisely expresses the view I have long since taken of the matter. I have arrived at these conclusions, because I, myself, possess the power of producing the snapping of the peroneus lungus over the malleolus externus, spoken of by M Schiff. I never had the satisfaction of being present at a rapping entertainment, but one night, at a social party, I made several *snaps* in the manner alluded to, when an intelligent gentleman, who had heard the *real* rappers, requested me to repeat the movements, and then stated that the sounds I produced were like the spirit raps, only not quite so loud. However, I can make myself heard in all parts of a large hall, and by practicing I could undoubtedly increase the *raps* in intensity. I have this power from my earliest recollection, and often amused my school-companions with my parformances. I can make the snaps faster than the tick of a watch, and in any position of the leg, whether supported or not. The act is entirely voluntary, and completely under my control, and the foot moved so little, that a person will not notice the slight jar it receives, unless his attention is particularly directed to the part. I can make the sound on the external malleolus of the right leg only, all my efforts to produce it with the left being vain.

Very respectfully,

H. J. BEYERLE, M. D.

Nippenose, Pa., Nov. 9, 1854.

